# Lifting the Veil of Morality: Choice Blindness and Attitude Reversals on a Self-Transforming Survey

**DOI:** 10.1371/journal.pone.0045457

**Published:** 2012-09-19

**Authors:** Lars Hall, Petter Johansson, Thomas Strandberg

**Affiliations:** 1 Lund University Cognitive Science, Lund University, Lund, Sweden; 2 Swedish Collegium for Advanced Study, Uppsala University, Uppsala, Sweden; CSIC-Univ Miguel Hernandez, Spain

## Abstract

Every day, thousands of polls, surveys, and rating scales are employed to elicit the attitudes of humankind. Given the ubiquitous use of these instruments, it seems we ought to have firm answers to what is measured by them, but unfortunately we do not. To help remedy this situation, we present a novel approach to investigate the nature of attitudes. We created a self-transforming paper survey of moral opinions, covering both foundational principles, and current dilemmas hotly debated in the media. This survey used a magic trick to expose participants to a reversal of their previously stated attitudes, allowing us to record whether they were prepared to endorse and argue for the opposite view of what they had stated only moments ago. The result showed that the majority of the reversals remained undetected, and a full 69% of the participants failed to detect at least one of two changes. In addition, participants often constructed coherent and unequivocal arguments supporting the opposite of their original position. These results suggest a dramatic potential for flexibility in our moral attitudes, and indicates a clear role for self-attribution and post-hoc rationalization in attitude formation and change.

## Introduction

Every day, thousands of opinion polls, corporate surveys, consumer panels, government feedback forms, and psychological rating scales are employed to elicit the attitudes of humankind. But what is that is being measured with these instruments? Given the ubiquitous use of survey and polling instruments it seems we ought to have firm answers to this fundamental question, but unfortunately we do not [1]–[4].

The typical approach to the issue is to focus on the predictive utility of the statements people make (irrespective of whether to call them attitudes, opinions, preferences, or evaluations). Hence, psychologists have long been troubling over the fact that what we say often does not predict what we do, and have tried different methodological twists to close the gap between attitudes and behavior [5]–[7]. Even less optimistically, in the debate over stated vs. revealed preferences, economists have often made wholesale dismissals of stated preferences in favor of market decisions [8]–[10].

Ideally, what researchers would like to have is a method that measured the propensity for consistency or change at the very moment of the poll (something that allows us to pre-emptively jump the attitude-behavior gap, so to speak). The standard way to approximate this goal is to complement a survey with meta-attitudinal judgements, such as perceived certainty or importance [11], [12]. These tools add to our predictive edge, but meta-attitudinal judgments have a tendency to fractionate into a grab bag of different factors and processes when closely scrutinized [13], [14]. In short, asking people to introspect and estimate their own propensity for change often assumes more self-awareness than is warranted from the evidence [4], [15]–[18]. Another possibility is to add some form of implicit measure, typically based on response latency [19], [20]. Again, this is helpful, but there is only so much information you can glean in a brief 100 msec reaction time window [21].

Yet, why do we have to conceive of attitude measurements primarily as reports, and not a form of interactive test or experiment? What would happen if we engaged more directly with the attitudes at hand, perhaps even challenged them? Using the phenomenon of *Choice Blindness* (CB) as a wedge, we have been able to separate the decisions of participants and the outcomes they are presented with. In aesthetic, gustatory and olfactory choices this has previously allowed us to demonstrate that participants often fail to notice mismatches between what they prefer and what they actually get (hence, being blind to the outcome of their choice), while nevertheless being prepared to offer introspective reasons for why they chose the way they did [22]–[24]. But what about the backbone of attitude research, all those surveys, panels and polls? If CB held across this domain it would create significant strain for our intuitive models of attitudes (in what sense can attitudes be real if people moments later fail to notice they have been reversed?), and provide us with a novel source for understanding prediction, persuasion, and attitude change (how will participants act after they have endorsed the opposite of what they just said?).

**Figure 1:**
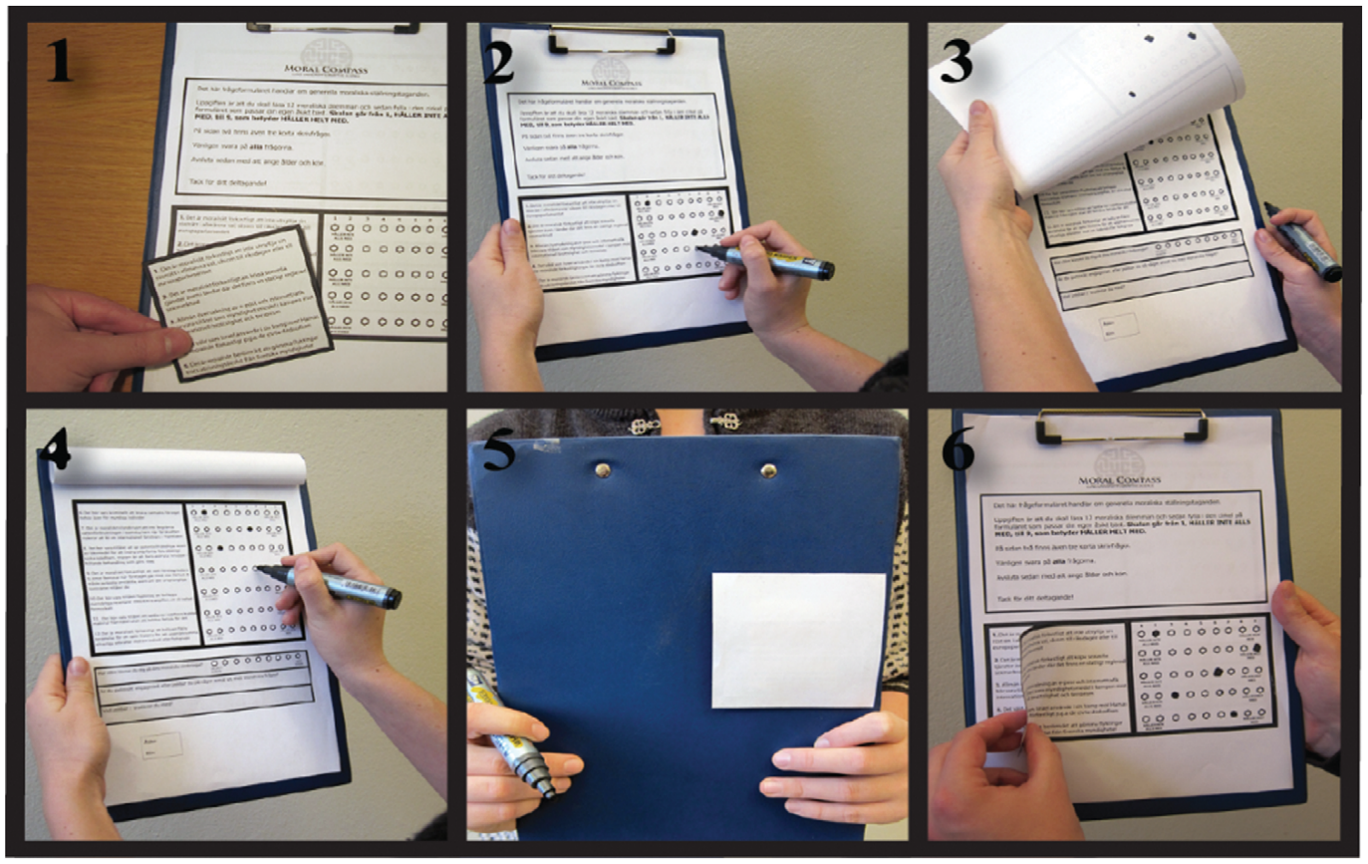
A snapshot of the choice procedure during a manipulation trial. (**1**) The questionnaire is attached to a clipboard, with the questions distributed over two pages. A paper slip with moral statements is attached to the first page of the questionnaire to conceal the same, but negated set of statements printed on the page. (**2**) The participants rate their agreement with the statements on the first page of the questionnaire and (**3**) they turn to the second page, and (**4**) rate their agreement with a second set of principles. (**5**) When the participants are asked to flip back the survey to the first page to discuss their opinions, the add-on paper slip from (**1**) now sticks to a patch of stronger glue on the backside of the clipboard, and remains attached there. This reveals the altered set of principles on the first page, and when the participants now read the manipulated statements the meaning has been reversed **(**in effect, the equivalent of moving the actual rating score to the mirror side of the scale). (**6**) During the debriefing, the experimenter demonstrates the workings of the paper slip to the participants, and explains how the manipulation led to the reversal of their position. See http://www.lucs.lu.se/cbq/for a video illustration of the method and the interaction between experimenter and participants.

To investigate these issues, we created a self-transforming paper questionnaire on moral attitudes using a methodology adapted from stage magic (see figure 1). The participants were given a survey on either foundational moral principles or moral issues hotly debated in the current media, and their task was to rate on a 9-point bidirectional scale to what extent they agreed or disagreed with each statement. After the participants had completed the questionnaire, we asked them to read aloud some of their answers from the first page, and to explain their ratings to us. However, unbeknownst to the participants, two of the statements they read aloud at this stage were actually the reverse of the statements they had originally rated – i.e. if the original formulation stated that “*large scale governmental surveillance of e-mail and Internet traffic ought to be forbidden as a means to combat international crime and terrorism.*”, it was now changed to “*large scale governmental surveillance of e-mail and internet traffic ought to be permitted as a means to combat international crime and terrorism.*”. As the rating was held constant but the direction of the statement was reversed, the participants’ original opinion was reversed as a consequence. Thus, this technique allowed us to expose participants to altered feedback about their previously stated attitude, and to create a situation in which we could record whether they were prepared to endorse and argue for the opposite moral view of what they stated only moments ago.

## Methods

### Participants

In total, 160 volunteers (100 female) participated in the study. Ages ranged from 17 to 69 years (M = 29.5, SD 10.8). We recruited the participants when they were walking through a park and asked them if they wanted to fill in a short survey about moral questions. All participants gave written informed consent to participate in the study, and all but 18 participants also agreed to have the interaction audio recorded.

### Ethics Statement

The study was approved by the Lund University Ethics board, D.nr. 2008–2435.

### Procedure and Materials

We presented the participants with a questionnaire containing 12 moral principles (condition one, N = 81) or statements describing 12 current moral issues (condition two, N = 79), and their task was to rate to what extent they agreed or disagreed with each statement on a 9-point bidirectional scale from 1 “completely disagree” to 9 “completely agree” (the midpoint of the scale allowed participants to be neutral or undecided about the issues). In the first condition we used fundamental moral principles adapted from Forsyth’s (1980) *Ethics Position Questionnaire*, such as “*It is more important for a society to promote the welfare of the citizens than to protect their personal integrity*”. In the second condition, we used concrete moral statements instantiating the principles from the first condition, e.g. “*Large scale governmental surveillance of e-mail and Internet traffic ought to be permitted as means to combat international crime and terrorism*.” (see table 1). The statements in condition two were picked to represent salient and important current dilemmas from Swedish media and societal debate at the time of the study, thus making it very likely that participants would have been exposed to prior information about the issues they were asked to express their attitudes on. In this way, we could create a contrast between foundational principles, what many suppose is the core of our moral beings, and the everyday manifestation of these principles in current issues, and investigate whether levels of abstraction would influence detection of the manipulations. Intuitively, we would expect abstract principles to allow for more exceptions and qualifications (a feature of abstractness as such), thus engendering lower levels of detection in this condition.

**Table 1:** List of moral principles and issues used for manipulation in condition one and condition two.

Original Principle	Reversed Principle	Original Issue	Reversed Issue
It is more important for a society toprotect the personal integrity of itscitizens than to promote their welfare	It is more important for a societyto promote the welfare of itscitizens than to protecttheir personal integrity	Large scale governmental surveillanceof e-mail and Internet traffic oughtto be forbidden as a means to combatinternational crime andterrorism.	Large scale governmental surveillanceof e-mail and Internet traffic ought tobe permitted as a means to combatinternational crime and terrorism.
Even if an action might harm theinnocent, it can still be morallypermissible to perform it	If an action might harm theinnocent, then it is not morallypermissible to perform it	The violence Israel used in the conflictwith Hamas is morally defensibledespite the civilian casualtiessuffered by the Palestinians.	The violence Israel use in the conflict withHamas is morally reprehensible becauseof the civilian casualties suffered by thePalestinians.
What is morally permissible oughtto be similar between differentsocieties and cultures	What is morally permissible oughtto vary between differentsocieties and cultures	It is morally defensible to purchasesexual services in democraticsocieties where prostitution is legaland regulated by the government	It is morally reprehensible to purchasesexual services in democratic societieswhere prostitution is legal and regulatedby the government
To be moral is to follow the rulesand regulations of the society,rather than weighing the positiveand negative consequences ofone’s actions	To be moral is to weigh the positiveand negative consequences ofone’s actions, regardless of therules and regulations of thesociety	It is morally deplorable to harborimmigrants when they have beendeclared illegal and scheduled toreturn to their home country bythe Swedish government	It is morally commendable to harborimmigrants when they have beendeclared illegal and scheduled to returnto their home country by the Swedishgovernment

In addition, we asked the participants to indicate how strong their moral opinions in general were, and if they were politically active or not, as well as their age and gender.

The questionnaire was attached to a clipboard, with the questions distributed over two pages. After completing the survey, we asked the participants to read aloud and discuss three of their ratings from the first page (randomly taken from a limited subset of the principles or statements), and also if it would be possible to audio-record this discussion. If the participants did not want to be recorded, the experiment leader took notes and made the necessary classifications immediately after the trial was completed. As previously explained, at this point two of the statements the participants read aloud had been reversed compared to the statements they had originally rated. When the participants had read the statement, we interjected and summarized their attitudes in a question by saying “so you don’t agree that [statement]?” or “so you do agree that [statement]?” to avoid any misunderstanding of what the rating implied. The reversal was achieved by attaching a lightly glued paper-slip on the first page of the questionnaire, containing the original version of the statements. The layout and shape of the attached slip allow it to blend in perfectly with the background sheet. When the participants folded the first page over the back of the clipboard, the paper-slip stuck on an even stickier patch on the backside of the questionnaire, thus revealing a new set of statements on the first page (see figure 1).

### Measures

All manipulated trials were categorized as either *corrected* or *accepted*. In the trials categorized as corrected, the participants either noticed the change immediately after reading the manipulated statement (spontaneous detection), or claimed in the debriefing session to have felt something to be wrong when reading the manipulated sentence (retrospective correction). In detail, we classified any trial as spontaneously detected if the participants showed any signs of having detected the change after reading the manipulated statement, e.g. if they corrected or reversed their rating to match their original position, or if they thought they must have misunderstood the question the first time they read it, etc. Most of the participants who immediately detected the manipulation also corrected the rating by reversing the position on the scale, i.e. had they rated their agreement to 1 (completely disagree) they changed it to 9 (completely agree) (although 10% of these trials were changed to a different number than the exact opposite). After the experiment, the participants were fully debriefed about the true purpose of the experiment. In this interview session we presented a series of increasingly specific questions about the experiment. Firstly we asked the participants in general what they thought about the questionnaire. Secondly, we asked if they had experienced anything as being strange or odd with the questionnaire. Finally, we showed them exactly how we had reversed some of the statements the second time, and asked whether they had felt a suspicion about anything like this during their responding. If at any point during this process they indicated they had felt something to be wrong when reading and responding to the manipulated statements, we asked them to point out which statements had been altered, and categorized these trials as retrospectively corrected. Consequently, in the trials categorized as accepted, there were no such signs of the participants having noticed that the opinions they argued for after the manipulation was the reversal of what they originally intended.

To create the most accurate representation of the number of detected trials we tried to create an experimental context with as little reluctance or possible awkwardness for the participants in correcting a manipulation as possible. In doing so, we stressed from the outset of the study that there were no time constraints for answering, that we had no moral or political agenda, and that we would not judge or argue their opinions in any way. Furthermore, the magic trick made the manipulation as such radically nontransparent, and thus it was near impossible for the participants to deduce the underlying intent of the study, and adapt their answers to please the experimenters. But at the same time, the design made it very easy and natural to correct any errors, as everyone is familiar with occasionally misreading or marking the wrong box on a form or survey. Similarly, in the debriefing session, our aim was to provide a sensitive and inclusive estimate of corrections, by giving the participants multiple opportunities with increasingly stronger cues to report any suspicions. If anything, we contend, the incentives of the final debriefing question encourages over-reporting of detections for those participants that do not want to admit to having accepted and argued for the reverse of their original rating. Our experience from prior studies of the CB phenomenon is that the category of detections and non-detections are sharply divided by the level of surprise and curiosity experienced by the participants in the debriefing sessions. It seems highly unlikely to us that participants are systematically withholding their feelings of detections, while at the same time acting as if they are genuinely surprised and curious about our explanation of the manipulations (e.g. see discussion of ‘choice blindness blindness’ in [23], [24]).

As the scale the participants used when rating their agreement with the moral principles or statements was bidirectional from 1 “completely disagree” to 9 “completely agree”, the midpoint (5) of the scale allowed participants to be neutral or undecided about the issues. As a consequence, in trials when the participants rated themselves to be neutral, the manipulated reversal of the principle or statement did not affect the participants’ stated opinion (i.e. they were still neutral). All such trials were removed from the analysis (36 of 320 M trials). An additional 13 M trials were removed due to technical failures in the manipulation process.

All the recordings of the participants’ argumentation were transcribed using the CHAT format, developed for the CLAN software. The direction and strength of the argumentation was estimated by three independent raters, and to avoid any bias in the classification of the verbal reports, all statements made by the experimenters, as well as any explicit mention of the direction of the rating, was removed from the transcripts.

## Results

### Corrected Trials

There were no differences in correction or acceptance rate when comparing the individual principles or statements in each condition. The result of each condition is therefore presented as a combined measure for the principles or statements being manipulated.

The majority of the manipulated trials remained undetected. In condition one, about one third of the trials was concurrently detected, and 8% of the trials were claimed to have been detected afterwards. In condition two, the concurrent detection rate was close to 50%, but very few participants claimed afterwards to have felt that something was wrong during the experiment (see table 2). Framing correction in terms of individuals instead of trials reveals that a remarkable 69% of all the participants accepted at least one of the two altered statement/rating relations. As hypothesized, the magic trick behind the self-transforming survey made sure virtually no participants noticed the manipulation as such. Instead, detections only took the form of self-corrections (“I must have misread”, “I must have marked the wrong end”, etc.).

**Table 2:** Distribution of corrected trials for manipulated trials in condition one and condition two.

	*Manipulated trials*			
	Spontaneous detection	Retrospective correction	Total	
*Condition*				
Condition 1: Moral principles	33.8%	10.6%	44.4%	
Condition 2: Moral issues	48.6%	1.4%	50.0%	
Total	41.3%	4.4%	47.2%	

The overall rating of the non-detected manipulated trials was notably high. Using a 9-point scale, the average rating was 2.8 or 7.2 depending on the direction of the rating, which means that the average ‘distance’ being manipulated when a statement was reversed was 4.4 units on the scale. This is evidence that the participants cared about the issues involved, and expressed seemingly polarized opinions about the manipulated issues they failed to detect. Interestingly, there were no significant difference in these rating averages between the two conditions (condition one = 4.5; condition two = 4.3). Thus, our intuition that abstract principles would involve more moderate attitudes, and engender less detection was not supported by the data. Instead, the result showed high involvement and equal levels of polarization in both the ‘abstract’ and ‘concrete’ condition.

The participants’ self-evaluation of the strength of their moral convictions was not correlated with correction (and neither was age, gender or time spent working on the questionnaire). Thus, participants who believed themselves to hold strong moral opinions in general were no more likely to correct the manipulations. However, the participants in condition two who classified themselves as politically active were more likely to concurrently detect the manipulations when comparing with the politically active participants in condition one (χ^2^_1_ = 5.72, p<.05, φ = .27). Controlling for this interaction effect, there was no difference in concurrent detection rate between condition one and two. Similarly, if we compare condition one and two with a combined measure of correction (concurrent correction, retrospective correction and reinterpretation of statement), we find no differences between the two conditions. Therefore, unless one is directly involved with the current dilemmas (as the politically active participants in condition two were), level of abstraction does not seem to affect levels of CB.

There was a positive relationship between the participants’ level of agreement and concurrent detection, i.e. the more the participants agreed or disagreed with a statement, the more likely they were to correct the manipulation. This was true in condition 1 (z = 2.97, p<.005, r = .25) as well as in condition 2 (z = 5.43, p<.0001, r = .45). Nevertheless, a full third (31.4%) of all manipulated trials rated at the endpoints of the scale (1 or 9) remained undetected, which shows that not even extreme levels of agreement or disagreement with statements guarantees detection. Additionally, in the dynamic of the magical reversal, participants expressing a highly polarized view were exposed to a more drastic alteration of their opinions than the more moderate ones (in effect turning a 1 into a 9, an 8 into a 2, etc.). Therefore, it is not clear whether participants with more polarized opinions are less amenable to change as such; perhaps there would have been no correlation between level of agreement and detection if the ratings of all the participants had been moved an equal distance on the scale (e.g. from 9 to 4, 8 to 3, etc).

### Accepted Trials

Equally as interesting as whether participants accept the reversed position is what they say when asked to explain the reasoning behind their ratings. From a common sense perspective, it seems reasonable to suspect that even if participants accept the manipulated position, they might still tend towards their original position in their verbal explanations. To examine if this was the case, we gave the transcribed verbal reports from non-manipulated trials (NM) and accepted manipulated trials (M) to three independent raters. The task for the raters was to blindly judge how strongly the verbal reports agreed or disagreed with a given moral principle or statement, using the same bi-directional 9-point scale as in the experiment. For the M trials, this measure reveals the degree to which the participants argue in support of their original or the reversed position. For example, if participants previously had stated that it is morally *deplorable* to harbor illegal immigrants, but the raters judge the verbal reports to rather indicate that they finds it morally *commendable* to harbor illegal immigrants, then this would be clear evidence that an attitude reversal has taken place.

There was a high level of inter-rater agreement between the three raters for the NM reports (r = .70) as well as for the M reports (r = .77), indicating that there are systematic patterns in the verbal reports that corresponds to certain positions on the rating scale for both NM and M trials. Even more interestingly, there was a high correlation between the raters estimate and the original rating of the participants for NM (r = .59) as well as for M reports (r = .71), which indicates that the verbal reports in the M trials do in fact track the participants rated level of agreement with the *opposite* of the initial moral principle or issue (for an illustration of this process and example reports, see figure S1, Supporting Online Material). In addition, this relationship highlights the logic of the attitude reversal, in that more modest positions result in verbal reports expressing arguments appropriate for the same region on the mirror side of the scale. And while extreme reversals more often are detected, the remaining non-detected trials also create stronger and more dramatic confabulations for the opposite position.

**Figure 2:**
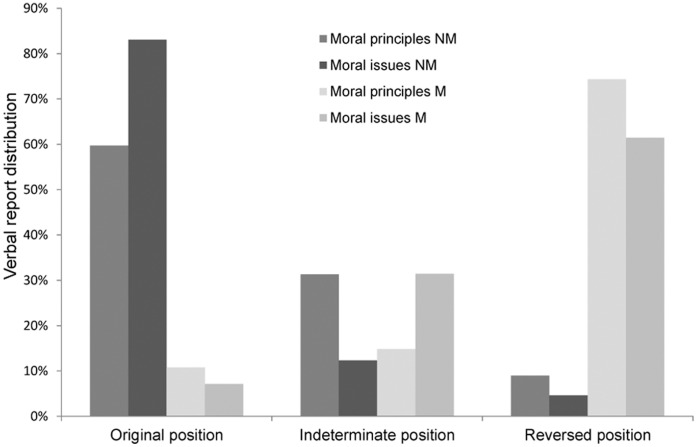
Classification of verbal reports in relation to rated agreement with a moral principle or statement, for NM and M reports, in condition one and condition two.

To visualize the difference between NM and M trials more clearly, we used the result from the independent raters to categorically classify whether the verbal reports supported the original or the reversed position. We classified all verbal reports, from both NM and M trials, into three different categories: i) *original position*; when the three raters uniformly agreed that the participants argued in the direction of their original position (that is, for the M trials, they reverted to their original position in the arguments after first having accepted the manipulation), ii) *indeterminate position*; when there was any level of disagreement between the raters as regards which position the participant supported, and iii) *reversed position*; when all three raters interpreted the verbal report as being in agreement with the opposite of the participants original position (see figure 2).

The result shows that the NM and M reports differ substantially: when there is no manipulation, what the participants say is primarily interpreted as supporting the original position, while the manipulated reports are seen as supporting the opposite of what they originally intended. The pattern of distribution for NM and M trials is almost mirror reversed, and consequently there is a highly significant difference when comparing the amount of original and reversed position trials for NM and M trials (χ^2^_1_ = 126.0, p<.0001, φ = .75). Expressed in terms of participants instead of trials, a full 53% of the participants argued unequivocally for the opposite of their original attitude in at least one of the manipulated trials.

## Discussion

It is easy to summarize the present study; participants express their moral opinions, then moments later many of them are blind to the mismatched outcome and endorse the opposite view. But it is considerably more difficult to explicate all the implications of it. If previously there was the trouble of stated attitudes often not translating into actions, now we have compounded this by showing that moral attitudes sometimes can be reversed moments after they are announced.

The most obvious suggestion to handle this problem would be to disqualify outright all opinions subject to CB as not real. Because how can it be a ‘real’ attitude if we moments later are prepared to endorse the opposite? Thus, absence of CB could be taken as a form of acid test for attitudes, a basic criterion for ‘attitudeness’. However, one would have to carefully consider the implications of such a criterion. In this study, we made an effort to choose a task that our participants would be knowledgeable about, and that would concern and engage them. To claim that half the Swedish population holds no articulated attitudes about the most visible moral issues in the current societal debate is a most uninviting conclusion to draw. Comparing this task to the ‘median attitude study’ in all the research fields and societal functions that trade in survey and rating data (which might solicit our opinions about anything from nasal decongestants, to boxaerobics, to diaper recycling, etc.) it seems the application of a CB-criterion for attitudeness would risk a monumental disqualification of current attitude measurements, and a widespread breakdown of survey psychology (including aspects of our own published work).

Another option would be to blame the scale instead of the participants; to suggest that the original rating simply failed to capture their ‘true’ attitudes. However, paradoxically we would then have to convince the participants themselves of the validity of this critique, because from their perspective they often argue their reversed position very convincingly (as seen in the correlation between their manipulated ratings, and the scores we blindly recreated from the transcripts, illustrated in figure 2). For all we know, had the participants not been debriefed at the end of the experiments, the attitudes we registered in the manipulation trials might had lived on to become persistent features of their ideology. In addition, this suggestion takes us down the same unattractive path of generalization as the previous one did. To brand our (in most regard typical) survey as meaningless would create very problematic consequences for scale psychology in general, where far more obscure ratings and far smaller differences than an average of 4.4 units on a 9-point scale routinely are taken to be of theoretical and practical value.

We are obviously hesitant to suggest any such calamitous consequences, but the alternatives presented above are at least worthy of serious consideration, and it seems to us that standard models of attitudes would have a difficult time explaining how attitude ratings could be reversed in the current way [25]–[27]; but see [28]–[30]? What is needed in order to navigate between the twin horrors of Scylla (‘most attitudes are not real’) and Charybdis (‘most attitude scales are meaningless’) is a systematic effort to relate our CB results to the issue of predictive utility and the attitude behavior gap. Regardless of whether the moral attitudes we measured were context dependently created, or stored evaluations inherent before the experiment (a distinction that in itself implies little about stability or change, [18], [31]), a CB snapshot might capture much of the same noise as is falling into the attitude-behavior gap, but without the need for repeated measurements from statement to behavior.

What remains to be seen is how our CB methodology relates to other measures of attitude strength. The use of meta-attitudinal judgments of confidence/certainty is exceedingly common in psychological research [32], [33]. But it seems improbable that the predictive edge we get from this would completely overlap or nullify CB manipulations. For example, in [22], we found no effect of the undetected manipulated trials on the expressed confidence of the participants in their choice of jam or tea, and in the current study, even at the extreme ends of our moral scale, a third of all manipulated trials remained undetected (thus leaving open the intriguing possibility of the meta-attitudinal judgments themselves being open for reversals). Similarly, how does CB relate to the central methodological concept of indifference curves in economic research on attitudes, which aim to find the point where participants are indifferent between options [34], [35]. It seems highly unlikely that CB would line up neatly with this point (participants would say some things matter, but not notice alterations, and say some things do not matter, but still reject alterations). This suggests we could instead work from the opposite direction to create a CB *difference curve* identifying alterations in the options that at least matter enough to be noticed. Also, if we move to response measures, classification of explicit and implicit attitudes often dissociate, but which (if any) factor is the most important driver for levels of CB? Would we exhibit more or less CB for attitudes that have been identified by implicit measures (such as the Implicit Association Test [20]? And would we be more willing to accept reversals that go in the direction of a dissociated or congruent implicit attitude?

As the discussion above shows, whatever theoretical perspective one brings to the discussion, the notion of opinions instantly reversing through CB creates considerable tension; specifically, for theories of moral attitudes, the current result seem to give support for models where moral decision or judgment is reached through intuition, and the reasons or arguments for the position are mainly constructed through post-hoc confabulation (as for example, the Social Intuitionist Model, [36], [37]). However, if we really had moral gut feelings, a form of spider sense tingling for the different options, it is difficult to envisage why so many of the participants would have failed to notice the reversed alternatives (it truly ought to have *felt* wrong to them). Thus, these reversals concerning both foundational principles and real world issues suggest that deliberation and argumentation (post-hoc, or not) play a more prominent role in moral judgments than acknowledged by the current crop of sentiment theories.

Framing it this way highlights the intriguing possibility that it might not always be considered an ideal to have the most minutely tuned attitudes, and to consistently notice all CB manipulations. Even if societal standards dictate a moral *ought* for citizens to educate themselves and form considered opinions about the issues covered in the current study, the complexity of the dilemmas are such that single-mindedness sometimes can invite suspicion (who am I to hold extreme attitudes about the righteousness of the different sides in the Palestine conflict, with its vast historical scope and complexities?). Similarly, while principles are supposed to be the very core of our moral beings, it might be something that only a rigid and legalistic mind actually *can* adhere to [38]. As argued by [39] with a simple shift of perspective from experimenter to participant, deplorable context ‘dependency’ turns into opportunistic context ‘sensitivity’. In this sense, the results could be seen as unmasking flexibility and openness to change that otherwise would be very difficult to demonstrate among the participants. Thus, while the experimenters remained completely neutral in the interaction, and presented no arguments or support for whatever position the participants presented, the unique dynamic of the experiment was that the participants (unwittingly) brought the full force of their argumentative powers to bear on *themselves* instead of others. This connects the current study to recent attempts at explaining the function of reasoning and argumentation as primarily being a means of convincing others that whatever conclusion I have reached is the correct one [40]. Furthermore, comparing our methodology to the classic debate about self-perception and dissonance reduction in social psychology [41], [42], CB gives us a novel and simple instrument to vary potential internal and external sources of inference in a dynamic account of attitude change. Hence, it would be interesting to see how the recorded attitude changes in the current study would compare with actual attempts at persuasion. Previous research has indicated how role-playing and consider-the-opposite inductions can alter attitudes [43], [44], but in this case the whole process would play out on an implicit level. Quite possibly, self-persuasion through CB could be more effective than interpersonal efforts at rational argumentation (Hall et al, unpublished data).

In summary, whether they are stated or revealed, inherent or constructed, stable or contextualized, the current study challenges our basic conception of what it means to express an attitude, and demonstrates a considerable malleability of everyday moral opinions. Future studies will determine how our CB methodology relates to established meta-attitudinal and implicit response time measures [45], [46], and to further explore the role for self-attribution and post-hoc rationalization in attitude formation and change.

## Supporting Information

Figure S1 **Sample verbal reports from undetected manipulated trials in relation to the** ***principle of harming the innocent*** **, and the** ***issue of governmental surveillance of e-mail and Internet traffic*** **(all reports have been translated from Swedish, and transcription notation has been removed for ease of reading).** Participants were presented with either an abstract principle or concrete moral issue, and then asked to indicate their attitude towards these on a scale from 1 (completely disagrees) to 9 (completely agrees). The figure in each cell of the table (a-h) shows the original rating of the participants as a filled red hexagon on the scale. In a manipulation trial, participants then face a negated principle or issue, which is the equivalent of moving their original rating to the mirror side of the scale. This dynamic is shown as a dotted red line ending in a X-marked hexagon in the figure. The verbal report the participants give at this point is shown in a speech bubble originating below the X-mark. Looking at the verbal reports it is evident that they present a much better fit to the manipulated side of the scale (the red X), than the original position (the red filling). This is further confirmed by the blue dot, which represents the attitude position the independent raters deemed most appropriate for the same report, when evaluated with no knowledge of the original position. Here, it can be seen that the blue dot consistently is placed on the same side of the scale as the red X, and much closer to it than to the original red filling.
(PDF)
