# Supplementary material for: Lifting the Veil of Morality: Choice Blindness and Attitude Reversals on a Self-Transforming Survey
Source: PLoS One. 2012 Sep 19;7(9):e45457. doi: 10.1371/journal.pone.0045457 (PMC3446893; doi:10.1371/journal.pone.0045457)
Supplement: Figure S1 — Sample verbal reports from undetected manipulated trials in relation to the principle of harming the innocent , and the issue of governmental surveillance of e-mail and Internet traffic (all reports have been translated from Swedish, and transcription notation has been removed for ease of reading). Participants were presented with either an abstract principle or concrete moral issue, and then asked to indicate their attitude towards these on a scale from 1 (completely disagrees) to 9 (completely agrees). The figure in each cell of the table (a-h) shows the original rating of the participants as a filled red hexagon on the scale. In a manipulation trial, participants then face a negated principle or issue, which is the equivalent of moving their original rating to the mirror side of the scale. This dynamic is shown as a dotted red line ending in a X-marked hexagon in the figure. The verbal report the participants give at this point is shown in a speech bubble originating below the X-mark. Looking at the verbal reports it is evident that they present a much better fit to the manipulated side of the scale (the red X), than the original position (the red filling). This is further confirmed by the blue dot, which represents the attitude position the independent raters deemed most appropriate for the same report, when evaluated with no knowledge of the original position. Here, it can be seen that the blue dot consistently is placed on the same side of the scale as the red X, and much closer to it than to the original red filling. (PDF) [file pone.0045457.s001.pdf]

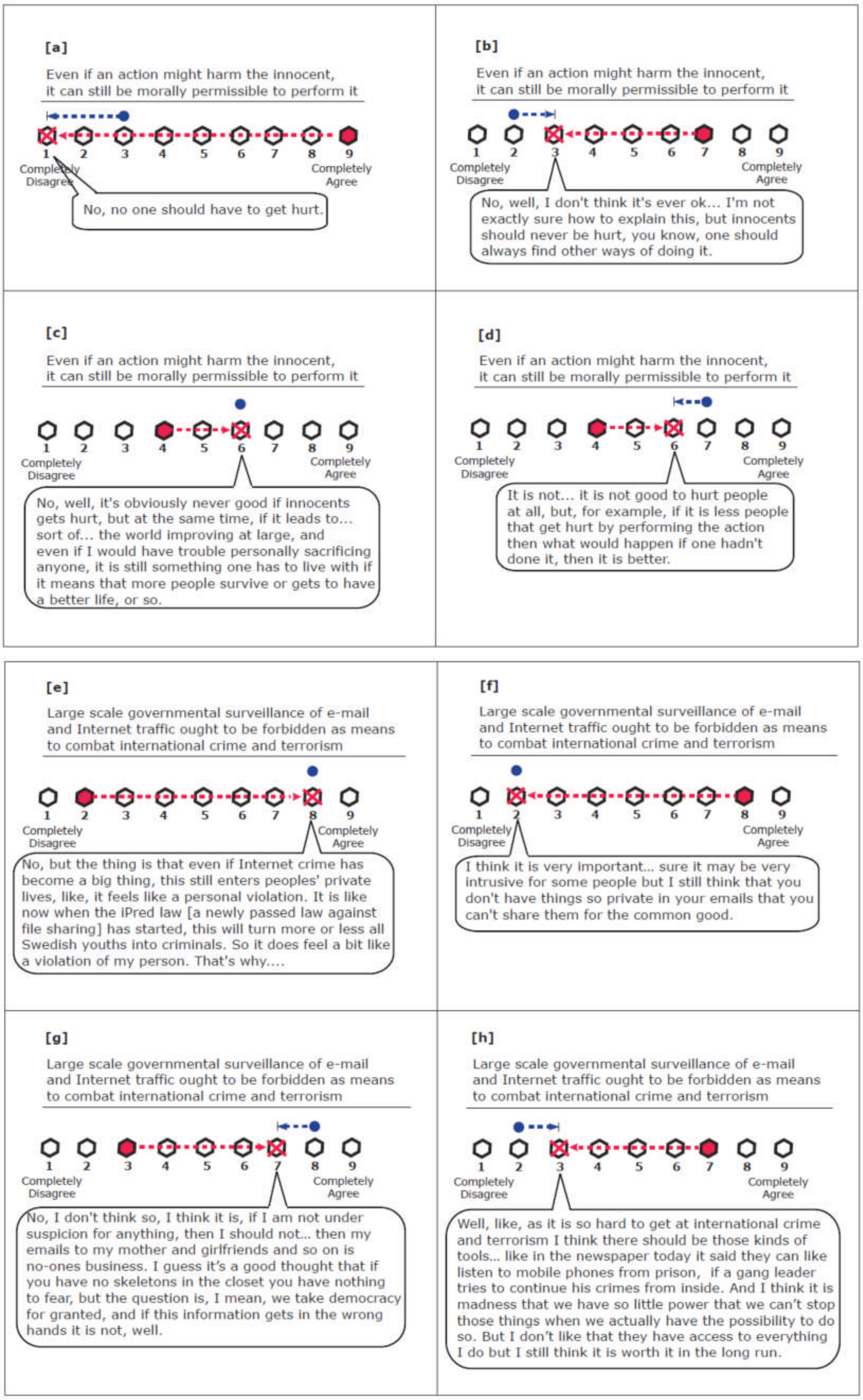
[a]
Even if an action might harm the innocent, it can still be morally permissible to perform it
1 2 3 4 5 6 7 8 9
Completely Disagree
Completely Agree
No, no one should have to get hurt.
[b]
Even if an action might harm the innocent, it can still be morally permissible to perform it
1 2 3 4 5 6 7 8 9
Completely Disagree
Completely Agree
No, well, I don't think it's ever ok... I'm not exactly sure how to explain this, but innocents should never be hurt, you know, one should always find other ways of doing it.
[c]
Even if an action might harm the innocent, it can still be morally permissible to perform it
1 2 3 4 5 6 7 8 9
Completely Disagree
Completely Agree
No, well, it's obviously never good if innocents gets hurt, but at the same time, if it leads to... sort of... the world improving at large, and even if I would have trouble personally sacrificing anyone, it is still something one has to live with if it means that more people survive or gets to have a better life, or so.
[d]
Even if an action might harm the innocent, it can still be morally permissible to perform it
1 2 3 4 5 6 7 8 9
Completely Disagree
Completely Agree
It is not... it is not good to hurt people at all, but, for example, if it is less people that get hurt by performing the action then what would happen if one hadn't done it, then it is better.
[e]
Large scale governmental surveillance of e-mail and Internet traffic ought to be forbidden as means to combat international crime and terrorism
1 2 3 4 5 6 7 8 9
Completely Disagree
Completely Agree
No, but the thing is that even if Internet crime has become a big thing, this still enters peoples' private lives, like, it feels like a personal violation. It is like now when the iPred law [a newly passed law against file sharing] has started, this will turn more or less all Swedish youths into criminals. So it does feel a bit like a violation of my person. That's why....
[f]
Large scale governmental surveillance of e-mail and Internet traffic ought to be forbidden as means to combat international crime and terrorism
1 2 3 4 5 6 7 8 9
Completely Disagree
Completely Agree
I think it is very important... sure it may be very intrusive for some people but I still think that you don't have things so private in your emails that you can't share them for the common good.
[g]
Large scale governmental surveillance of e-mail and Internet traffic ought to be forbidden as means to combat international crime and terrorism
1 2 3 4 5 6 7 8 9
Completely Disagree
Completely Agree
No, I don't think so, I think it is, if I am not under suspicion for anything, then I should not... then my emails to my mother and girlfriends and so on is no-ones business. I guess it's a good thought that if you have no skeletons in the closet you have nothing to fear, but the question is, I mean, we take democracy for granted, and if this information gets in the wrong hands it is not, well.
[h]
Large scale governmental surveillance of e-mail and Internet traffic ought to be forbidden as means to combat international crime and terrorism
1 2 3 4 5 6 7 8 9
Completely Disagree
Completely Agree
Well, like, as it is so hard to get at international crime and terrorism I think there should be those kinds of tools... like in the newspaper today it said they can like listen to mobile phones from prison, if a gang leader tries to continue his crimes from inside. And I think it is madness that we have so little power that we can't stop those things when we actually have the possibility to do so. But I don't like that they have access to everything I do but I still think it is worth it in the long run.

Figure S1 online material. Sample verbal reports from undetected manipulated trials in relation to the *principle of harming the innocent*, and the *issue of governmental surveillance of e-mail and Internet traffic* (all reports have been translated from Swedish, and transcription notation has been removed for ease of reading). Participants were presented with either an abstract principle or concrete moral issue, and then asked to indicate their attitude towards these on a scale from 1 (completely disagrees) to 9 (completely agrees). The figure in each cell of the table (**a-h**) shows the original rating of the participants as a filled red hexagon on the scale. In a manipulation trial, participants then face a negated principle or issue, which is the equivalent of moving their original rating to the mirror side of the scale. This dynamic is shown as a dotted red line ending in a X-marked hexagon in the figure. The verbal report the participants give at this point is shown in a speech bubble originating below the X-mark. Looking at the verbal reports it is evident that they present a much better fit to the manipulated side of the scale (the red X), than the original position (the red filling). This is further confirmed by the blue dot, which represents the attitude position the independent raters deemed most appropriate for the same report, when evaluated with no knowledge of the original position. Here, it can be seen that the blue dot consistently is placed on the same side of the scale as the red X, and much closer to it than to the original red filling.
